# Stepping-stones and dispersal flow: establishment of a meta-population of Milu (*Elaphurus davidianus*) through natural re-wilding

**DOI:** 10.1038/srep27297

**Published:** 2016-06-07

**Authors:** Daode Yang, Yucheng Song, Jianzhang Ma, Pengfei Li, Hong Zhang, Mark R Stanley Price, Chunlin Li, Zhigang Jiang

**Affiliations:** 1 Institute of Wildlife Conservation, Central South University of Forestry and Technology, Changsha, 410004, China; 2 Eastern Dongting Lake National Nature Reserve, Yueyang, Hunan, 414000, China; 3 Northeast Forestry University, Harbin, 150040, China; 4 Hubei Shishou Milu National Nature Reserve, Shishou, 434400, China; 5 Wildlife Conservation Research Unit, University of Oxford, The Recanati-Kaplan Centre, Tubney House, Abingdon Road, Tubney OX13 5QL, UK; 6 College of Environment and Resource, Anhui University, Hefei, 230601, P.R. China; 7 Institute of Zoology, Chinese Academy of Sciences, Beijing, 100101, China

## Abstract

The Milu (Père David’s deer, *Elaphurus davidianus*) became extinct in China in the early 20^th^ century but was reintroduced to the country. The reintroduced Milu escaped from a nature reserve and dispersed to the south of the Yangtze River. We monitored these accidentally escaped Milu from 1995 to 2012. The escaped Milu searched for vacant habitat patches as “stepping stones” and established refuge populations. We recorded 122 dispersal events of the escaped Milu. Most dispersal events occurred in 1998, 2003, 2006 and 2010. Milu normally disperse in March, July and November. Average dispersal distance was 14.08 ± 9.03 km, with 91.41% shorter than 25 km. After 5 generations, by the end of 2012, 300 wild Milu were scattered in refuge populations in the eastern and southern edges of the Dongting Lake. We suggest that population density is the ultimate cause for Milu dispersal, whereas floods and human disturbance are proximate causes. The case of the Milu shows that accidentally escaped animals can establish viable populations; however, the dispersed animals were subject to chance in finding “stepping stones”. The re-wilded Milu persist as a meta-population with sub-populations linked by dispersals through marginal habitats in an anthropogenic landscape.

Why and how animals disperse are hot topics in behavioral ecology^1^. Dispersal is affected by population density, resource competition, mating competition, in-breeding avoidance and fluctuation in habitat carrying capacity^2^,^3^,^4^. For reintroduced species, dispersal after release refers to the movement of individuals from the site of release to their temporary or permanent habitats^5^. Dispersal is often observed during the initial period after the release of reintroduced individuals, as a searching process in an unknown environment^6^,^7^. Linklater & Cameron^8^ classified animal dispersal into temporary dispersal and permanent dispersal: in the former the dispersed individuals return to the original population or site, while in the latter the dispersed individuals leave the original population or site permanently^8^. If released individuals cannot adapt to the environment, then the reintroduction may fail^9^,^10^. High mortality of juveniles, or low density or low fecundity during the initial period after release may reduce the success of reintroduction^9^,^10^. Thus, finding suitable habitat, range expansion and establishing a population are key stages for colonization by reintroduced individuals. These can also be indicators of progress for a reintroduction project^11^.

Due to habitat loss and fragmentation, wild animals living in isolated, small populations face a higher risk of extinction^12^. In anthropogenic landscapes wild animal habitats are often fragmented into patches surrounded by areas that are not suitable habitat^13^,^14^. To disperse in anthropogenic landscapes, animals may use “stepping stones”, which describe habitat patches providing food and shelter for the dispersers within unsuitable habitat. Thus, stepping stones facilitate movements of individuals and increase connectivity between habitat patches^15^. When individuals form breeding herds in the stepping stones and breed for at least one generation, such habitat patches may be referred as “refuges”; in contrast, small habitat patches only serve as “stepping-stones” for further dispersal.

Milu (*Elaphurus davidianus*, also called Père David’s deer) is endemic to China. Milu are morphologically adapted to wetlands^16^,^17^,^18^,^19^. They were once widely distributed in the lower and middle reaches of the Yangtze River, but became extinct in the country due to habitat loss, hunting and climate change around the turn of the 20^th^ century^20^,^21^. In 1985, Milu were reintroduced from England to Beijing Milu Park, China^22^. In 1991, Hubei Shishou Milu National Nature Reserve (SMNNR, 112.23°E, 29.49°N), 15.67 km^2^ in area, was established on an island in the middle reach of the Yangtze River. A total of 64 Milu were transferred to SMNNR from the Beijing Milu Park in 1993 and 1994. Five years later, in May 1998, thirty-six Milu escaped from SMNNR during a heavy flood of the Yangtze River; these runaway Milu established populations and bred successfully in the wild^17^. The total number of naturally re-wilded Milu and their descendants exceeded 500 by the end of 2009, but the re-wilded Milu population declined during an outbreak of disease from mid-February to mid-March 2010. Their pattern of dispersal and consequent distribution resulted by chance in establishment of a meta-population, a situation totally different from the managed releases of captive-bred Milu to the Yellow Sea coast in the Dafeng Milu Nature Reserve, Jiangsu Province, China^23^. In the former, no human management was imposed on the escaped Milu. While researchers have studied the feeding, breeding, habitat, behavior, population dynamics of the reintroduced and enclosed Milu^17^,^19^,^24^,^25^,^26^,^27^,^28^, little work has been done on naturally re-wilded Milu^18^.

In this study, we first describe the development process and current distribution of the re-wilded Milu meta-population. We then document the frequency and distance of dispersal of the escaped Milu from SMNNR to characterize the process. Third, we explore factors such as population density, flooding and human disturbance that might be the causes of the dispersal of Milu. Finally, we discuss the management of naturally re-wilded Milu.

## Study Area

The study area is located in the sub-tropical monsoon zone of China. Average annual relative humidity is about 80%, annual precipitation is 1,200–1,450 mm, and annual evaporation is 1,470 mm. Annual mean temperature ranges 16.5–17.0 °C; the maximum temperature was 38.4 °C while minimum temperature was −14.9 °C, while 240–315 days every year are frost-free^24^. Dongting Lake (3,968 km^2^) links with the Yangtze River and serves as a buffer for the floods in the Yangtze River. The water catchment area of Dongting Lake is 262,800 km^2^. Annual variation in the water level of the lake is about 12.9 m^29^, with the Yangtze River flooding between May and October. Average altitude in the study area is 40 m above sea level^30^. The lake shores are fertile lands for producing rice^31^. In the study area, vegetation is dominated by reeds and sedges and non-native, planted trees like *Populus euramevicana.* Wetlands in the study area are home to hundreds of thousands of water birds. Local wild mammals are mainly shrews: *Crocidura attenuata* and *Chimarogale himalayica*; rodents: *Microtus fortis*, *Micromys minutes*, *Apodemus agrarius*, *Rattus rattus*, *R. niviventer*, *R. losea*, *R. tanezumi*, *Niviventer fulvescens*, and small carnivores: *Mustela sibirica davidiana*, *M. kathiah* and *Melogale moschata ferreogrisea*; other species include the hare *Lepus tolei*, and hedgehog *Erinaceus europaeus.* Rural people in the study area make a living from farming, fishing and reed farming. Reeds are harvested in the study area for making paper, and reed farming is an important industry in the local economy. Each reed farm has up to 5 reed management stations for managing the reed fields.

The Yangtze River transects the study area of 19,801 km^2^. The study area, which is called “the country of fish and rice”, has a long history of human occupation. Human population density exceeds 300 people/km^2^ and may be as high as 2,800 people/km^2^ in urban areas, each of which is much higher than the national average, 143 people/km^2^ 32.

## Methods

Establishment of the meta-population of dispersed Milu was monitored by tracking the escaped and dispersing Milu in the field. From the re-introduction of Milu to SMNNR in 1993–1994 to May 1998, only few Milu tried to escape from SMNNR. During this period, Milu searched for suitable habitat outside the SMNNR because the north side of the reserve was not fenced; thus Milu could escape from SMNNR. However, these Milu dispersed only a short distance. During that period, whenever we detected a Milu escape event or there was a report of Milu escaping, we would try to find the escaped Milu and recorded the time, location of the escape event and number of Milu which escaped, if the escaped Milu returned, then we noted it as a temporary dispersal, otherwise the dispersal is noted as a permanent dispersal. But, when the Yangtze River was heavily flooded in May 1998, a large herd of Milu escaped and swam crossed the Yangtze River. It was impossible to drive the escaped Milu back in the swamp area. From that time on, SMNNR staff took on the duty of monitoring the escaping Milu, and we developed a monthly survey scheme to monitor the dispersed Milu; we called this the “routine survey”. We also made instantaneous surveys when we received any observations of dispersed Milu; in such cases, we rushed directly to the reported sites.

We carried out routine surveys every mid-month; 3–4 investigators formed a survey group and surveyed along the route: Yangbotan(YBT) -Sanheyuan(SHY) -Nannianzi(NNZ)–East Dongting Lake. When, after 2006, Milu dispersed further to south Dongting Lake and west Dongting Lake, we extended our routine survey routes to these areas. Surveys were mostly carried out from motor cycles or bicycles in YBT, SHY and NNZ. When dispersed Milu were reported or seen, surveys were carried out on foot. We used boats along the Zhuzihe River(ZZH), landing at the each reed farm management station, where we interviewed the workers and surveyed the area around the reed managing station. We used a boat in Hongqihu Lake(HQH) when the water level was high, and then landed and surveyed on lakeshore. When the water level was low in the lake, the area was surveyed on foot.

Milu is the only large deer in the study area, so it and its traces like fecal pellets and hoof prints are easily identified. When signs of Milu were discovered during a field survey, we looked for Milu living at that site. If we found Milu still in the area, we then monitored the herd to document the number of individuals, and the date when the Milu started to disperse to next “stepping stone”. We recorded the date, whether through a routine survey or instantaneous survey, GPS coordinates of the start point and destination of dispersal, the number of Milu (with the numbers of male, female or juveniles when possible), the habitat, and whether direct or indirect survey methods were used (Appendix 1).

By interviewing local farmers, we learnt of the arrival date of Milu at any site, when the Milu started the next dispersal, and hence the duration of stay there. If only signs, but no living Milu, were discovered, the times when Milu arrived and left a site were obtained by interviewing local farmers.

To avoid disturbing Milu we searched with binoculars an area of 100 m. on either side of our track. When Milu were in open areas or during winter, they were counted.

On the other hand, it was impossible to see the Milu in reed fields when reeds were high and they would move away on detecting human presence as farmers were driving the re-wilding Milu away from reed farms and rice paddies. In such situations, and when there was evidence that Milu had left the area, indirect methods had to be used. We used evidence such as hoof prints, fecal pellets, hairs and grazing signs or bedding traces to determine where the Milu had come from. Fresh hoof prints in paddy fields were used to record numbers and the dispersal direction.

In addition to the rangers of the SMNNR and Hunan East Dongting Lake National Nature Reserve (EDLNNR), a total of 8 graduate students under the supervision of the first author worked on the Milu for their theses or dissertations in the area, and all participated in the field surveys during the period.

When all the Milu left one site to disperse to another, we treated this as a single dispersal event. We recorded the date and origin of dispersal, and looked for the subsequent “stepping stones” of the dispersed animals. If the Milu successfully found a new “stepping stone” and permanently left the original habitat, then we defined the dispersal as “effective dispersal”. Total dispersal frequency is denoted as all the dispersals during one year, while effective dispersal frequency is denoted as all the effective dispersals occurring within a year. Dispersal distance is the linear distance between the origin of dispersal (SMNNR) and the next stepping stone or the farthest site the Milu reached.

We studied the relationship between population density and dispersal frequency from 2002–2011 in the relatively isolated SHY sub-population. This area is a reed farm, surrounded by the Yangtze River, and by dikes and farmlands. We directly counted the number of Milu individuals in SHY in spring and winter when reeds were short. The population density was calculated as:


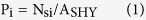


Where: P_i_ is the population density in the SHY sub-population in year i; N_si_ is number of Milu in the SHY sub-population in spring of year I and A_SHY_ is area of SHY (provided by the local reed farm).

Dispersal frequency was calculated as:


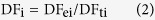


Where: DF_i_ is the dispersal frequency in the SHY sub-population; DF_ei_ is the effective dispersal frequency in the SHY sub-population in the year and DF_ti_ is the total dispersal frequency in the SHY sub-population in the year.

### Analyses

We explored whether dispersals of Milu were linked to its population density, human interference, floods or food shortage in winter. We chose the SHY sub-population as an example to test the hypothesis that dispersal in the naturally re-wilded Milu is driven by its population density. The SHY sub-population occupies a relatively contained reed field; it borders the Yangtze River on the east and north and is separated by dikes from farmlands, towns and villages. Milu in SHY seldom crossed the dikes except in floods. Because of this situation, we could calculate the density of this sub-population.

The sub-populations of YBT, ZZH and HQH occupy areas with incomplete water boundaries or ones in which water levels drop so that Milu can come and go. Hence, the SHY sub-population was used to explore the relationship between Milu density and dispersal.

Correlation between Milu population density and dispersal frequency was analyzed with Pearson correlation. Dispersal rates between different periods were tested with the Mann-Whitney U test. Differences in dispersal distances of different Milu sub-populations were analyzed with ANOVA. Significant levels were set at *P* < 0.05. Data were presented as Mean ± SD. We used the coordinates of the origin of dispersal and of the “stepping stone” to calculate dispersal distance with Google Earth. The map of Milu dispersal was drawn with DIVA-GIS (Version 7.1.7, http://www.diva-gis.org/Download); geographic data were downloaded from http://www.diva-gis.org/Datadownload.

## Results

The group sizes of Milu in 75 of 122 recorded dispersals were directly counted in field, carried out in winter or spring when the vegetation was short (Appendix 1). The recorded number of dispersing Milu ranged from one to 56 (Appendix 1). For the remaining 47 cases, the local farmers only remember the month in which Milu arrived. In 17 cases, outside routine surveys, the local farmers only recalled the year of the Milu dispersal took place, but all dispersals were validated by traces of Milu at previously occupied sites.

**Figure 1:**
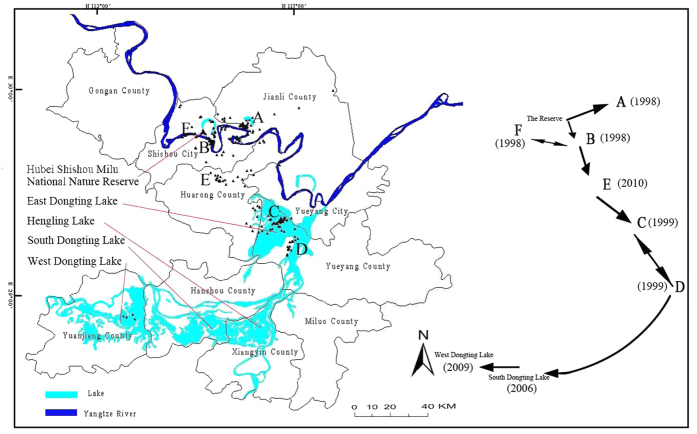
The stepping stones and dispersal flow of naturally re-wilding Milu in the Dongting Lake area. Milu escaped from the Hubei Shishou Milu National Nature Reserve (SMNNR) and found their first batch of stepping stones: Yangbotan wetland (YBT, **A**) on the north of the Yangtze River,Sanheyuan Wetland (SHY, **B**) and Nannianzi Reeds Farm (NNZ, **F**) on the south of the Yangtze River in 1998; NNZ is a temporary refuge for Milu from SHY; there are still Milu between NNZ and SHY. The free ranging Milu found new stepping stones- Zhuzihe riverside wetland (ZZH, **C**) and Hongqihu Wetland (HQH, **D**) in 1999 while some Milu went back north to Shengfengxiang (SFX, **E**) from SHY during a disease outbreak to establish a sub-population there. YBT, SHY, ZZH and HQH are the main re-wilded Milu sub-populations named sequentially from the time of its establishment. The Milu continued to disperse south of the Dongting Lake and established a sub-population in South Dongting Lake which was a source sub-population in 2006 for Milu inside the South Dongting Lake National Nature Reserve (SDLNNR). In 2009, the Milu dispersed to west Dongting Lake and established the West Dongting Lake sub-population. This figure was created by Yucheng Song using the DIVA-GIS 7.1.7 (http://www.diva-gis.org/download). It is a free software. The data of geographic information were downloaded from http://www.diva-gis.org/datadown.

Since 1995, Milu dispersed from the SMNNR to six counties and cities in Hubei and Hunan provinces, including Jianli County, Hubei Province and Huarong County, Yueyang County, Yueyang City, Yuanjiang County, Hanshou County of Hunan Province (Fig. 1).

Based on the dispersal frequency in different periods, movements of the escaped Milu could be divided into an exploratory dispersal stage, a stable dispersal stage and a fast dispersal stage:

### Exploratory dispersal stage (1995–1997)

The Milu were introduced to SMNNR in 1993 and 1994 in two batches. The reintroduced Milu started to wander outside the reserve almost immediately upon release there. The exploratory dispersal stage of the Milu in SMNNR was from 1995 to 1997. Because the southern border of SMNNR was not fenced until 1998, two, one and another one Milu left SMNNR and swam across the Yangtze River in 1995, 1996 and 1997, respectively, and they appeared in the wetland of SHY on the south bank of the Yangtze River. However, all these dispersals were temporary for the runaway Milu explored the environment outside the reserve and returned to SMNNR.

### Stable dispersal stage (1998–2002)

In May 1998 the Yangtze River experienced a heavy flood with a chance of occurrence of once in a hundred years, Eight dispersals of Milu were recorded in that year. Of these, five dispersals were permanent dispersals and responsible for the foundations of sub-populations in YBT and SHY. The high water levels caused 36 Milu of the population of 84 to escape from SMNNR. 10 of them stayed in the YBT wetland (29°44′~50′ N; 112°41′~49′ E, A in Fig. 1) on the north bank of the Yangtze River and 26 Milu swam across the Yangtze River and settled down in SHY. Thereafter, the escaped Milu started to breed in YBT and SHY.

YBT is the only refuge for escaped Milu on the north of the Yangtze River. By the end of 2012 the Milu population size in YBT reached about 100, occupying a patch of 21.19 km^2^ wetland, which is surrounded by farmlands and villages in the east, west and north sides, and the Yangtze River on the south. Milu in YBT had a relatively stable range area. However, as a large part of the wetland would be submerged under water during the flood season from May to October each year, a small number of Milu left the refuge during floods and swam to south of the Yangtze River in 1998.

SHY (5.39 km^2^ in area) is a flood buffer zone, which connects with the Yangtze River on the east and north; the west and south sides are surrounded by dikes and farmlands. SHY is 16 km from YBT and located south of the Yangtze River, and has served as a “stepping stone” for the dispersed Milu moving from north to south of the Yangtze River since 1998.

As the population of the dispersed Milu in the SHY increased and available habitat became submerged during floods, the Milu left the SHY refuge and moved to EDLNNR (145.40 km^2^). In the spring of 1999, some Milu which had dispersed from SHY moved to the third and fourth “stepping stones” of the ZZH riverside wetland and HQH wetland (C and D in Fig. 1, 28°59′~29°23′ N; 112°42′~113°1′ E) and established the EDLNNR sub-population.

### Fast expansion stage (2003–2012)

Altogether, 102 dispersals of individuals or groups occurred during the period from 2003 to 2012. Most dispersals were recorded in 2003, 2006 and 2010. In general, the frequency of dispersal increased through the period.

The population size of the Milu meta-population fluctuated. When the SHY wetland was submerged during floods each year, some Milu in SHY emigrated to the NNZ Reed Farm. Most of the emigrant Milu returned to SHY after the flood season but a few Milu stayed on NNZ. Due to a shortage of food in SHY in December every year, some Milu in SHY went back to NNZ to search for food, returning to SHY in the next spring. The population size of SHY increased from 99 in 2003 to 223 in 2009; then the population started to decline, so there were only 167 Milu in SHY by the end of 2012. 77 Milu lived in YBT wetland at the end of 2012. The EDLNNR sub-population was periodically augmented with Milu dispersing from SHY and its population size increased to 68–80 at the end of 2012.

During an outbreak of disease from mid-February to mid-March 2010, the number of Milu in SHY declined from 223 in 2009 to 167 at the end of 2012. Disease was implicated as the main source of mortality, but occasionally, Milu calves drowned during dispersal when they tried to cross water. Further, during the rut, Milu stags were also strangled by the fishing nets set up in the lake and ponds.

As the population densities of dispersed Milu increased and available habitat area fluctuated in the “stepping stones” during floods, the Milu, especially those in the SHY and EDLNNR, started to expand their ranges. When population density in SHY reached 30.61 Milu/km^2^ in spring of 2006, some Milu moved southward to the wetlands in EDLNNR. Dispersed Milu presumably used the wetlands of EDLNNR as “stepping stones”; some Milu dispersed south and west of the Dongting Lake areas. Milu in YBT on the northern bank of the Yangtze River also dispersed at higher rates than those in SHY and EDLNNR; however, those dispersals from YBT were all temporary dispersals. Presumably, no suitable habitats were found by the dispersed Milu when they moved out of YBT.

The first Milu in south Dongting Lake area were sighted in 2006. Three years later, Milu were sighted in west Dongting Lake area (28°47′~29°07′ N; 111°57′~112°17′ E). However, no stable Milu populations were established in those “stepping stones” in the south and west Dongting Lake areas. In 2010, Milu density in SHY reached 41.37 Milu/km^2^; a disease broke out in the population and caused heavy mortality. At this time, 24 Milu left SHY wetland and emigrated to SFX (E in Fig. 1) and established a refuge sub-population there.

**Figure 2:**
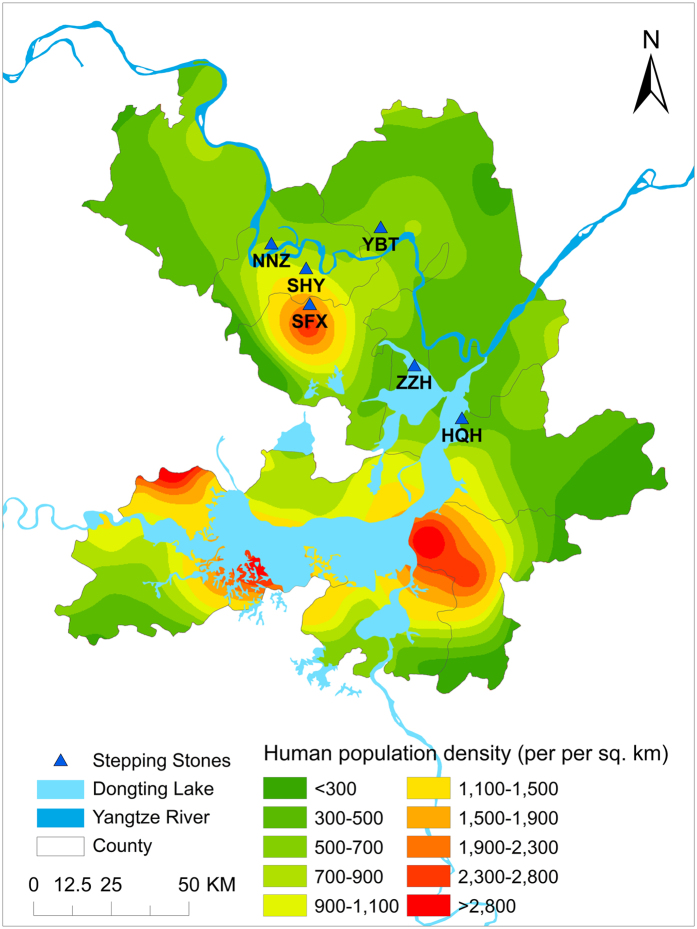
Human population density and the stepping stones of the naturally re-wilded Milu. Note the naturally re-wilded Milu establish sub-populations in areas with low human densities. In the figure: YBT stands for Yangbotan wetland, SHY for Sanheyuan wetland, ZZH for Zhuzihe riverside wetland, HQH for Hongqihu wetland, SFX for Shengfengxiang and NNZ for Nannianzi Reeds Farm. SFX is an agricultural area with intense human activity; here a small herd of dispersed Milu stayed amongst poplar trees on the tops of hills during daytime and then went down the hills to graze in the rice paddies at dawn and dusk, from where they were frequently driven away by farmers. This figure was created by Chunlin Li, using ArcGIS 9.3 (http://www.esri.com/) with ESRI 2008 Authorization number ECP280340058.

The most northern recorded location of dispersed Milu was 14 km from the origin of dispersal, SMNNR, while the most southern recorded location was 49.69 km distant. More Milu dispersed to the south than to the north (Fig. 2). Milu avoided areas with intense human activities such as farmlands, villages, and towns except where the Milu could find shelter as in the woods in SFX with less human activities (Fig. 2).

**Figure 3:**
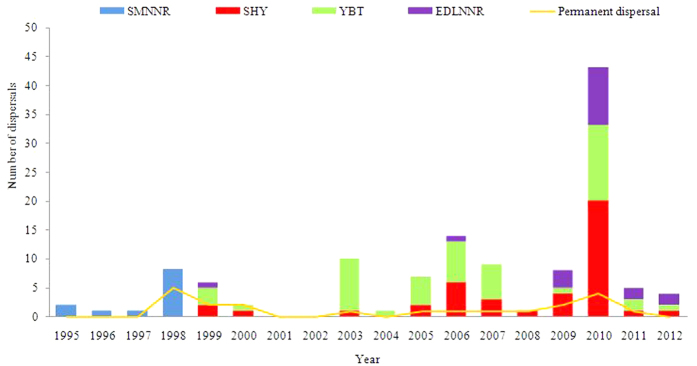
Dispersal frequency and effective dispersals of all the naturally re-wilded Milu since 1995. The number of permanent dispersals peaked in 1998 during the rare heavy flood, and then during a disease outbreak in Sanheyuan (SHY) sub-population in 2010. The numbers of dispersed Milu are recorded in Appendix 1.

We recorded 122 dispersals from 1995 to 2012 (Fig. 3). 1998, 2003, 2006 and 2010 were the peak years of dispersal, with 8 (6.56%), 9 (7.38%), 14 (11.48%) and 43 (35.25%) dispersal events, respectively. However, there were only 21 effective dispersals out of 113 temporary dispersals (Fig. 3). The frequency of dispersal in 1998–1999 was lower than the following dispersal peak year, but effective dispersal in 1998–1999 was higher than the following dispersal peak year (Mann-Whitney U, *U*  = −2.012, *P* < 0.05). Effective dispersal rates were 62.50%, 40.00%, 0, 7.14% and 9.30% in 1998, 1999, 2003, 2006 and 2010, respectively.

### Impact of population density

**Figure 4:**
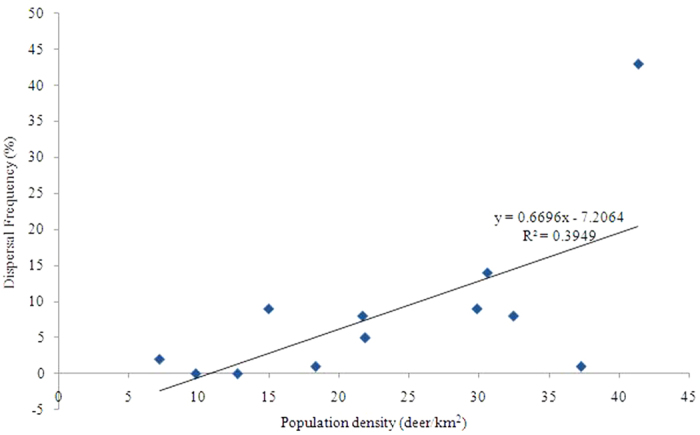
Relationship between the percentage of dispersals each year as percentage of all dispersals between 1995 and 2011 and population density in the Sanheyuan (SHY). The numbers of dispersed Milu are recorded in Appendix 1.

Using escaped Milu in SHY as an example, dispersal frequency positively correlated with population density from 2000–2011 (Pearson correlation: *r* = 0.628, n = 12, *P* = 0.029). Yearly dispersals of Milu increased as population density increased (Fig. 4).

### Interference from human activity

**Figure 5:**
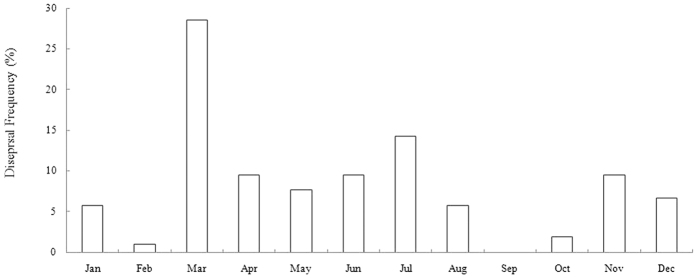
The percentage of dispersals each year to total dispersals as percentage of all dispersals between 1995 and 2011 of the re-wilded Milu in different months (1995–2012).

30(25%), 15(12%) and 10(8%) dispersals of Milu occurred in March, July and November respectively (Fig. 5). Although food was abundant in spring, Milu may leave the “stepping stones” in spring due to interference from human activities.

Grazing and trampling on reed shoots in spring by Milu in the reed fields damages reeds and causes loss of production; the reed farmers in SHY and YBT drive the Milu away from reed fields in March each year. In March 2003 the farmers carried out a large scale operation to drive the re-wilded Milu away and forced three groups of re-wilded Milu to leave,, which explains the high frequency in this month in Fig. 5. Veterinarians and reserve staff went to SHY to investigate dead Milu in March 2010, which also disturbed the Milu and caused 10 groups of re-wilded Milu to leave SHY to SFX, which accounted for 25% of total dispersals in the year.

SFX is an agricultural area with intense human activity; here dispersed Milu stayed amongst poplar trees on the tops of hills during daytime and then went down the hills to graze in the rice paddies at dawn and dusk, and so were frequently driven away by farmers. Consequently, less than 10 re-wild Milu lived in SFX by the end of 2012, others having returned to SHY. This small sub-population in a refuge area is unlikely to be viable in the longer term.

### Floods

Seasonal floods from the Yangtze River from May to July every year threatened the local survival of wild Milu. Floods submerge the “stepping stones” of Milu, forcing them to leave and to find new “stepping stones” or move to higher places in neighboring areas (Fig. 5).

## Discussion

The Milu gave up safety in the nature reserve for free-living in a heavily populated region where the human density is much higher than the national average. Why did escaped Milu not go back to SMNNR where they would be under protection, and free of human disturbance? Presumably, Milu prefer a mobile existence in which they make choices of habitat. Dispersal reduces local population density, and consequently reduces competition for food and presumably reduces the chance of suffering disease epidemics. The dispersal of Milu may have multiple causes.

### Ultimate cause

Dispersed Milu migrated through the anthropogenic landscape across a cluster of “stepping stones”. In the longer term, refuge populations might face local extirpation or loss of genetic diversity^33^,^34^. Dispersing individuals, driven by local high population density, would potentially benefit the gene pool. Additionally, dispersal would reduce the competition and grazing pressure on habitat.

Population density often interacts with fluctuations in environment. Abnormal and adverse environmental conditions may intensify the impact of population density^35^,^36^. Within large herbivores white rhinoceros (*Ceratotherium simum*)^37^, elk (*Cevus elaphus*)^38^ and black tailed deer (*Odocoileus hemionus*)^39^ all exhibit increases in individual dispersal distances as population density increases. In this study, when population density increased, Milu tended to disperse from SHY. Therefore, we suggest that population density is the ultimate cause for Milu dispersal.

### Proximate causes

Proximate causes of Milu dispersal include human interference, floods and seasonal changes in habitat.

Wetlands in Dongting Lake area are flood buffer zones where there is low human activity and which provide seasonal refuges for the Milu between annual spring floods. Historically, hunting and other human activities may have been the main cause of local Milu extinction in the wild^25^,^40^. Even after being bred in captivity for centuries, Milu still remain vigilant for natural predators^41^. In this study, grazing and trampling by the escaped Milu damaged reed fields and rice paddy; Milu are then always driven away and forced to disperse by local farmers. In anthropogenic landscapes, human interference is a factor driving Milu from one habitat patch to another.

Annual rainfall in the middle and lower Yangtze River drainage is as high as 2000 mm, with most rain falling in spring and summer. Dongting Lake is a buffer of the Yangtze River, storing water when the river floods in spring and summer and discharging it when water in the river is low in autumn and winter. Available habitat in wetlands around the lake for the Milu depends on the water level during lake floods. Floods may reduce available food and shelter; the population densities of Milu in the “stepping stones” increased during floods as most parts of the “stepping stones” were submerged under water. In due course this forced the Milu to leave and migrate to farmlands despite their high human interference. Farms and villages occupy the higher land in the study area^28^. Reed fields are semi-natural vegetation and low in human activity outside the reed-harvesting season, and therefore provide temporary shelter for Milu; Milu occupied these reed beds and grazed on reed shoots until they were driven away by farmers. Consequently, Milu moved between the SHY wetland and NNZ.

### Dispersal frequency

Milu dispersal from SMNNR was an exploratory process. Similar processes are found in rodents, herbivores and carnivores^6^,^7^,^42^,^43^,^44^. The effectiveness of dispersal of Milu in the stable stage of dispersal was higher than that in the other two stages of dispersal. Presumably, there were empty “stepping stones” available during the stable stage of dispersal. After a dispersal peak (Fig. 3), less Milu dispersed which suggests that when some individuals had dispersed permanently, the population density was thus reduced or those animals that were prone to dispersal had left the population.

### “Stepping stones”

“Stepping stones” are links between habitat patches^45^. The effectiveness of a stepping stone relies not only on the area and quality of habitat, but is influenced by the composition of substrates of surrounding patches^15^. The composition and type of patches influence the dispersal speeds of animals^46^.

Any suitable habitat patch is a potential stepping stone for animal dispersal. The straight-line distance between SHY and EDLNNR is 52.29 km; Milu stopped over at a series of “stepping stones” to reach EDLNNR. In contrast to animals using corridors to disperse, Milu adopted the dispersal strategy of stepping stones, and thus moved through habitat barriers^47^ in an anthropogenic landscape.

When a “stepping stone” is large enough or has no human interference for a period, then it may become a “refuge” for the dispersed Milu. Although such refuges are often marginal lands for people and may be of low habitat quality, they are important for maintaining re-wilding Milu populations. When population density increases or when food resources are depleted or habitat area is reduced during floods, competition among individuals is likely to intensify; some Milu will then move and search for new “stepping stones”.

In the wetlands of Dongting Lake, some lowlands only emerge seasonally from water; thus those wetlands are only seasonal refuges for dispersing animals. Establishment of the SHY and EDLNNR sub-populations and the decline of the SFX “refuge” sub-population indicated that the type, quality and area of the habitat patches actively influence the establishment and persistence of dispersed Milu populations.

### Meta-population

The naturally re-wilded Milu formed a meta-population. The movement of individuals between sub-populations is usually a one-way dispersal of individual Milu from an established sub-population to a relatively new sub-population; at the same time this is a movement from an established sub-population of high density to a new sub-population of lower density of Milu. The extirpation of some local populations and the establishment of new local populations are both characteristic of meta-populations^48^.

The key for successful dispersal in fragmented habitat is the animals’ dispersal ability and the availability of suitable patches. Consequently, the size and quality of habitat patches will affect meta-population dynamics^49^. Milu might search for available habitat patches in all directions, but the location of the next stepping-stone determines effective dispersal. Additionally, the distance between two stepping-stones may also affect successful dispersal. Assuming there is no stepping-stone at SFX, establishing a local population at East Dongtong Lake may be delayed due to the great distance between the EDLNNR sub-population and other source populations; SFX is located between SHY and East Dongtong Lake at distances of 19 km and 36 km respectively from them. The distance between SHY and East Dongtong Lake is 52.20 km.

### Implications for conservation

There is great seasonal variation in rainfall along the course of the Yangtze River. Consequently due to major seasonal differences in water level, there are vast wetlands, and lakes such as Dongting Lake which are connected to the Yangtze River and have wide lakeshore wetlands which absorb floods. Such seasonal, flooded wetlands are of marginal economic value to people, but their ecological value in buffering floods is significant. Lowlands along the Yangtze River and Dongting Lake are also available habitats for the Milu during the dry season of the year. Further nature reserves have recently been established to protect the riverside and lakeshore wetlands and their biodiversity^50^.

To restore species in the wild, we should not only rely on deliberately releasing animals into their past range. Sometimes, accidentally escaped animals can successfully establish viable populations, as in the case of the Milu. Provided the escape takes place in former range of the species, the natural re-wilding process is likely to favor the fittest individuals.

Free-ranging animals in anthropogenic landscapes are subject to the chance of finding “stepping stones” or “refuges” during dispersal. The Milu’s network of stepping stones, refuges and associated dispersals suggest a meta-population structure with presumed dynamic gene flows as well as source and sink sub-populations. Such a meta-population structure should increase resilience against future extinction. Conservation planning and management of existing nature reserves could attract the Milu and they would then exploit the seasonally fluctuating wetland habitats to greater extent, which could be promoted through conservation planning and management in the existing nature reserves. If so, then Milu can be expected to persist in an area with a long history of human exploitation that is still densely populated by human beings.

## Additional Information

**How to cite this article**: Yang, D. *et al.* Stepping-stones and dispersal flow: establishment of a meta-population of Milu, (*Elaphurus davidianus*) through natural re-wilding. *Sci. Rep.* **6**, 27297; doi: 10.1038/srep27297 (2016).

## Supplementary Material

Supplementary Information

## References

[b1] Ochiai K. & Susaki K. Causes of natal dispersal in monogamous ungulate, the Japanese serow, Capricorniscripus. Anim. Behav. 73, 125–131 (2007).

[b2] Bowler D. E. & Benton T. G. Causes and consequences of animal dispersal strategies: relating individual behaviour to spatial dynamics. Biol. Rev. 80, 205–225 (2005). 15921049 10.1017/s1464793104006645

[b3] Long E. S., Diefenbach D. R., Rosenberry C. S. & Wallingford B. D. Multiple proximate and ultimate causes of natal dispersal in white-tailed deer. Behav. Ecol. 19, 1235–1242 (2008).

[b4] Long E. S., Diefenbach D. R., Wallingford B. D. & Rosenberry C. S. Influence of roads, rivers, and mountains on natal dispersal of white-tailed deer. J. Wildl. Manage. 74, 1242–1249 (2010).

[b5] Dunham K. M. Dispersal pattern of mountain gazelles *Gazella gazelle* released in central Arabia. J. Arid. Environ. 44, 247–258 (2000).

[b6] Larkin J. L., *et al.* Influences on release-site fidelity of translocated elk. Restor. Ecol. 12, 97–105 (2004).

[b7] Steury T. D. & Murray D. L. Modeling the reintroduction of lynx to the southern portion of its range. Biol. Conserv. 117, 127–141 (2004).

[b8] Linklater W. L. & Cameron E. Z. Social dispersal but philopatry reveals incest avoidance in a polygynous ungulate. Anim. Behav. 77, 1085–1093 (2009).

[b9] Armstrong D. P. & Seddon P. J. Directions in reintroduction biology. Trends. Ecol. Evol. 23, 20–25 (2008). 18160175 10.1016/j.tree.2007.10.003

[b10] Le Gouar J. D., Mihoub J. & Sarrazin F. Dispersal and habitat selection: behavioural and spatial constraints for animal translocations. Reintroduction Biology: Integrating Science and Management (eds. Ewen J. G., Armstrong D. P., Parker K. A. & Seddon P. J. ), 138–164, (Wiley-Blackwell, Oxford, 2012).

[b11] Bright P. W. & Smithson T. J. Biological invasions provide a framework for reintroduction: selecting areas in England for pine marten releases. Biodivers. Conserv. 10, 1247–1265 (2001).

[b12] Brown J. H. & Kodric-Brown A. Turnover rates in insular biogeography, effect of immigration on extinction. Ecology 58, 445–449 (1977).

[b13] Singleton P. H., Gaines W. L. & Lehmkuhl J. F. Landscape Permeability for Large Carnivores in Washington: A Geographic Information System Weighted-Distance and Least-Cost Corridor Assessment, (Research Paper PNW-RP-549, Forest Service, USDA, 2002).

[b14] Boscolo D., Candia-Gallardo C., Awade M. & Metzger J. P. Importance of interhabitat gaps and stepping-stones for Lesser Woodcreepers (*Xiphorhynchus fuscus*) in the Atlantic Forest, Brazil. Biotropica 40, 273–276 (2008).

[b15] Simberloff D., Farr J. A., Cox J. & Mehlman D. W. Movement corridors: Conservation bargains or poor investments? Conserv. Biol. 6, 493–504 (1992).

[b16] Li C., Jiang Z., Zeng Y. & You Z. A note on environmental elements as essential prerequisites for behavioral expression: A case study of Père David’s deer. Anim. Behav. Sci. 102, 353–359 (2007).

[b17] Yang D. D. *et al.* Population dynamics of the Père David’s deer *Elaphurus davidianus* in Shishou Milu National Nature Reserve, Hubei Province, China. Acta Zoologica Sinica 53, 947–952 (In Chinese with English Abstract) (2007).

[b18] Wu B., Ji Y., Wang J. & Qin P. The annual habitat selection of released Père David’s Deer in Dafeng Milu National Nature Reserve. Acta Ecologica Sinica 31, 225–232 (2011).

[b19] Xu J. N. *et al.* Behaviors of Pére David’s deer harem master during its rutting period in Shishou County of Hubei Province, China. Chinese J. Ecol. 32, 1277–1282 (In Chinese with English Abstract) (2013).

[b20] Cao K., Qiu L., Chen B. & Niao B. Chinese Milu. (In Chinese) (Xuelin Press, Shanghai, China, 1988).

[b21] Cao K. Selection of suitable area for re-introduction of wild Père David’s deer in China. Deer of China: Biology and Management (eds. Ohtaishi. N. & Sheng H. L. ), 297–300 (Elserver Science, B. V. Amsterdam, the Netherlands, 1993).

[b22] Jiang Z. *et al.* Reintroduction and recovery of Père David’s deer in China. Wildl. Soc. Bull. 28, 681–687 (2000).

[b23] Hu H. & Jiang Z. Experimental release of Père David’s deer in Dafeng Reserve, China. Oryx 36, 196–199 (2002).

[b24] Yang D. D. Reintroducing Père David’s Deer in the Dongting Lake Region, China: History, Practice and Feasibility. PhD thesis. (In Chinese with English Abstract) (Northeast Forestry University, Harbin, China, 2004).

[b25] Yang D. *et al.* Causes of endangerment or extinction of some mammals and its relevance to the reintroduction of Père David’s deer in the Dongting Lake drainage area. Biodivers. Sci. 13, 45–461 (In Chinese with English Abstract) (2005).

[b26] Zou S. J., Song Y. C., Yang D. D. & Li P. F. Winter bed-site microhabitat selection by Père David’s deer (*Elaphurus davidianus*) in Hubei Shishou Milu National Reserve, South-central China. Chinese. J. Ecol. 32, 899–904 (In Chinese with English Abstract) (2013).

[b27] Song Y. C. *et al.* Regulation of free-ranging Milu population in Shishou, Hubei, China: a density-dependent decrease in birth rate. Biodiver. Sci. 2015, 23, 33–40 (In Chinese with English Abstract) (2015).

[b28] Song Y. C. *et al.* Sex-biased dispersal in naturally re-wild Milu in the Dongting Lake Region, China. Acta Ecologica Sinica 35, 4416–4424 (In Chinese with English abstract) (2015).

[b29] Xue L. Q., Hao Z. C., Liu X. Q. & Li Y. K. Numerical simulation and optimal system scheduling on flood diversion and storage in Dongting basin, China. Procedia. Environ. Sci. 12, 1089–1096 (2012).

[b30] Lai X. J., Huang Q. & Jiang J. H. Wetland inundation modeling of Dongting Lake using two-dimensional hydrodynamic model on unstructured grids. Procedia Environmental Science 13, 1091–1098 (2012).

[b31] Du Y. *et al.* Lake area changes in the middle Yangtze region of China over the 20th century. J. Environ. Manage. 92, 1248–1255 (2011). 21220184 10.1016/j.jenvman.2010.12.007

[b32] State Statistical Bureau. *Statistical Yearbook of China*. Beijing: State Statistical Bureau of P. R. China (2013). (In Chinese) http://www.stats.gov.cn/tjsj/ndsj/Retrieved on Feb. 2, (2016).

[b33] Kramer-Schadt S., Revilla E., Wiegand T. & Breitenmoser U. Fragmented landscapes, road mortality and patch connectivity: modelling influences on the dispersal of Eurasian lynx. J. Appl. Ecol. 41, 711–723 (2004).

[b34] Zeng Y., Jiang Z. & Li C. Genetic variability in relocated Père David’s deer (*Elaphurus davidianus*) populations—Implications to reintroduction program. Conserv. Genet. 8, 1051–1059 (2007).

[b35] Bonenfant C. *et al.* Empirical Evidence of Density-Dependence in Populations of Large Herbivores. Adv. Ecol. Res. 41, 313–357 (2009).

[b36] Matthysen E. Density-dependent dispersal in birds and mammals. Ecography 28, 403–416 (2005).

[b37] Owen-Smith R. N. Megaherbivores: The Influence of Very Large Body Size on Ecology, (Cambridge University Press, Cambridge, 1988).

[b38] Catchpole E. A. *et al.* Sexual dimorphism, survival and dispersal in red deer. J. Agr. Biol. Envir. St. 9, 1–26 (2004).

[b39] Bunnell F. L. & Harestad A. S. Dispersal and dispersion of black-tailed deer: models and observations. J. Mammal. 64, 201–209 (1983).

[b40] Frid A. & Dill L. M. Human-caused disturbance stimuli as a form of predation risk. Conserv. Ecol. 6, 11 (2002).

[b41] Li C. *et al.* Do Père David’s deer lose memories of their ancestral predators? PLos One 6, 1–6 (2011). 10.1371/journal.pone.0023623 PMC3160898 21887286

[b42] Forbes S. H. & Boyd D. K. Genetics structure and migration in native and reintroduced Rocky Mountain wolf populations. Conserv. Biol. 11, 1226–1234 (1997).

[b43] Moehrenschlager A. & MacDonald D. W. Movement and survival parameter of translocated and resident swift fox *Vulpes velox*. Anim. Conserv. 6, 199–206 (2003).

[b44] Preatoni D. *et al.* Conservation of brown bear in the Alps: space use and settlement behavior of reintroduced bears. Acta. Oecol. 28, 189–197 (2005).

[b45] Taylor P. D., Fahrig L., Henein K. & Merriam G. Connectivity is a vital element of landscape structure. Oikos 68, 571–573 (1993).

[b46] Ricketts T. H. The matrix matters: effective isolation in fragmented landscapes. Am. Nat. 158, 87–99 (2001). 18707317 10.1086/320863

[b47] Haddad N. M. Corridor length and patch colonization by a butterfly, Junonia coenia. Conserv. Biol. 14, 738–745 (2000).

[b48] Day J. R. & Possingham H. P. A Stochastic Meta-population Model with Variability in Patch Size and Position. Theor. Popul. Biol. 48, 333–360 (1995).

[b49] Howe R. W., Davis G. J. & Mosca V. The demographic significance of “sink” population. Biol. Conserv. 57, 239–255 (1991).

[b50] Ministry of Environmental Protection. Chinese Fifth National Report on the Implementation of the Convention on Biological Diversity. Beijing: China Environmental Publishing House (2014).

