# Supplementary material for: Stepping-stones and dispersal flow: establishment of a meta-population of Milu (Elaphurus davidianus) through natural re-wilding
Source: Sci Rep. 2016 Jun 7;6:27297. doi: 10.1038/srep27297 (PMC4895148; doi:10.1038/srep27297)
Supplement: Supplementary Information [file srep27297-s1.pdf]

Title: **Stepping-stones and dispersal flow: establishment of a meta-population of Milu (*Elaphurus davidianus*) through natural re-wilding**

Running title: **Natural re-wilding of escaped Milu**

Daode Yang[1], Yucheng Song[1, 4], Jianzhang Ma[2], Pengfei Li[3], Hong Zhang[4], Mark R Stanley Price[5], Chunlin Li[6] & Zhigang Jiang[7*]

[1]Institute of Wildlife Conservation, Central South University of Forestry and Technology, Changsha, 410004, China,

[2] Northeast Forestry University, Harbin, 150040, China,

[3]Hubei Shishou Milu National Nature Reserve, Shishou, 434400, China,

[4] Eastern Dongting Lake National Nature Reserve, Yueyang, Hunan, 414000, China,

[5]Wildlife Conservation Research Unit, University of Oxford, The Recanati-Kaplan Centre, Tubney House, Abingdon Road, Tubney OX13 5QL, UK

[6]College of Environment and Resource, Anhui University, Hefei, 230601, P.R. China,

[7] Institute of Zoology, Chinese Academy of Sciences, Beijing, 100101, China.

Appendix 1 Field records of Milu dispersed from the Shishou Milu National Nature Reserve (SMNNR)

Time [1]	R/I [2]	Start point	Site abbreviation	Coordinates of start point		Destiny	Coordinates of destiny		Distance (km)[3]	Habitat	No. of Milu [4]	Methods
1995-5	J	SMNNR	SMNNR	29.791589	112.548242	SHY	29.742483	112.59835	7.31	Reeds and swamps	1	Hoof prints
1995-03-13	J	SMNNR	SMNNR	29.791589	112.548242	Xiaohe Farm	29.782217	112.59252	4.44	Farmlands	2	Direct observation, fecal pellets
1996	J	SMNNR	SMNNR	29.791589	112.548242	SHY	29.736872	112.59719	7.19	Reeds and swamps	1	Hoof prints
1997	J	SMNNR	SMNNR	29.791589	112.548242	SHY	29.736185	112.59706	7.7	Reeds and swamps	1	Hoof prints
1998	R	SMNNR	SMNNR	29.791589	112.548242	SHY	29.742366	112.59041	6.66	Reeds and swamps	U	Hoof prints, fecal pellets
1998	R	SMNNR	SMNNR	29.791589	112.548242	YBT	29.818485	112.7238	17.35	Reeds and swamps	U	Hoof prints, fecal pellets
1998	R	SMNNR	SMNNR	29.791589	112.548242	EDLNNR	29.437181	112.80679	46.46	Reeds and swamps	U	Hoof prints, fecal pellets
1998	R	SMNNR	SMNNR	29.791589	112.548242	Xinhezhou	29.757233	112.67259	12.52	Riverine meadow, reeds	U	Hoof prints, fecal pellets
1998	R	SMNNR	SMNNR	29.791589	112.548242	Jiaoziyuan	29.907659	112.43559	16.96	Farmlands	U	Hoof prints, fecal pellets
1998	R	SMNNR	SMNNR	29.791589	112.548242	Yanduancun	29.530661	112.74657	34.69	Farmlands	U	Hoof prints, fecal pellets
1998-11	R	SMNNR	SMNNR	29.791589	112.548242	SFX	29.559328	112.62764	26.68	Woods, farmlands	U	Hoof prints, fecal pellets
1998	R	SMNNR	SMNNR	29.791589	112.548242	Jianli	29.801828	112.86993	31.21	Riverine meadow	U	Hoof prints, fecal pellets

1999	R	Sanheyuan	SHY	29.742483	112.59835	Xinhezhou Reed Farm	29.754418	112.67828	9.34	Reeds	U	Hoof prints, fecal pellets
1999-3	J	Zhuzihe	ZZH	29.364575	112.913967	HQH	29.377793	112.93377	11.4	Reeds and swamps	U	Hoof prints, fecal pellets
1999-6	R	Sanheyuan	SHY	29.742483	112.59835	Xinzhouwaitan	29.754418	112.67828	11.81	Reeds and swamps	U	Hoof prints, fecal pellets
1999-3	R	Yangbotan	YBT	29.818485	112.723797	Jiaoziyuan Villege	29.918129	112.44044	16.96	Farmlands	U	Hoof prints, fecal pellets
1999	R	Yangbotan	YBT	29.818485	112.723797	Guangyici	29.873912	112.88717	16.85	Farmlands	U	Hoof prints, fecal pellets
1999	R	Yangbotan	YBT	29.818485	112.723797	Maoshizhen	29.902131	113.00498	28.94	Farmlands	U	Hoof prints, fecal pellets
2000	R	Sanheyuan	SHY	29.742483	112.59835	NNZ	29 °46'43.17"	112 °28'12.78"	12.65	Reeds and meadows	U	Hoof prints, fecal pellets
2000	R	Yangbotan	YBT	29.818485	112.723797	Xinhezhouwaitan	29 °44'40.68"	112 °41'23.20"	2.83	Reeds and meadows	U	Hoof prints, fecal pellets
2003-12	R	Sanheyuan	SHY	29.742483	112.59835	NNZ	29.746903	112.58846	12.89	Reeds	U	Hoof prints, fecal pellets
2003-03-25	J	Yangbotan	YBT	29.818485	112.723797	Xiaohe Farm	29.787833	112.60522	13.77	Meadows	13(7 M;5F)	Direct observation
2003-03-25	J	Yangbotan	YBT	29.818485	112.723797	Qianzitou	29.84025	112.59307	15.23	Meadows	4 M	Direct observation
2003-03-26	J	Yangbotan	YBT	29.818485	112.723797	Liuheyuan	29.807617	112.59925	14.3	Meadows	11(7 M, 3F)	Direct observation
2003-04-16	R	Yangbotan	YBT	29.818485	112.723797	Xiaohe Farm	29.787833	112.60522	13.98	Reeds	13(2J)	Direct observation
2003-04-16	R	Yangbotan	YBT	29.818485	112.723797	Qianzitou	29.844228	112.58012	15.23	Meadows	4 M	Direct observation
2003-07-	R	Yangbotan	YBT	29.818485	112.723797	Tianxingzhou	29.810667	112.63527	10.9	Woods	30	Direct observation

18												
2003-08-12	R	Yangbotan	YBT	29.818485	112.723797	Tianxingzhou	29.81175	112.65403	9.01	Woods	50	Direct observation
2003-11-11	R	Yangbotan	YBT	29.818485	112.723797	Tianxingzhou	29.803793	112.63489	10.98	Woods	13	Direct observation, bedding trace
2004-01-02	J	Yangbotan	YBT	29.818485	112.723797	Beinianzi	29.812733	112.49537	24.46	Farmlands	2 M	Direct observation
2005-03-05	R	Yangbotan	YBT	29.818485	112.723797	Xiaohe Farm	29.788067	112.60497	14.09	Reeds	2 M	Direct observation
2005-03-05	R	Sanheyuan	SHY	29.742483	112.59835	Taohuashan	29.676756	112.69065	11.7	Farmlands and woods	U	Direct observation
2005-06-17	R	Yangbotan	YBT	29.818485	112.723797	Youyi Farm	29.874367	112.61917	14.14	Farmlands	U	Direct observation
2005-11-19	R	Sanheyuan	SHY	29.742483	112.59835	Jiaoshanghe	29.644667	112.56358	10.7	Farmlands	1 M	Direct observation
2005-11-30	J	Yangbotan	YBT	29.818485	112.723797	Nanhezhou	29.801733	112.65293	9.21	Reeds	2 M	Direct observation
2005-12-01	J	Yangbotan	YBT	29.818485	112.723797	Dagonghu	29.858917	112.53325	21.37	Meadows, farmlands	1 M	Direct observation
2005-12-05	R	Yangbotan	YBT	29.818485	112.723797	Nanhezhou	29.801733	112.65293	13.27	Reeds	2 M	Direct observation
2005-12-31	J	Sanheyuan	SHY	29.742483	112.59835	NNZ	29.801733	112.65293	12.78	Reeds	U	Direct observation
2005-12-31	J	Yangbotan	YBT	29.818485	112.723797	Jiaoshanghe	29.644667	112.56358	10.85	Reeds	U	Direct observation
2006-03-	R	Sanheyuan	SHY	29.742483	112.59835	Lianxinyuan	29.680571	112.61309	6.65	Farmlands	20	Direct observation

09												
2006-03-10	R	Sanheyuan	SHY	29.742483	112.59835	Dongsheng Villege	29.711169	112.51386	6.92	Farmlands	1 F	Direct observation
2006-03-13	R	Sanheyuan	SHY	29.742483	112.59835	Tiaoguan	29.694589	112.64393	6.87	Farmlands	30	Direct observation
2006-03-15	R	Zhuzihe	ZZH	29.364575	112.913967	Tuanzhou	29.318816	112.78357	9.45	Reeds	30	Direct observation
2006-03-15	R	Yangbotan	YBT	29.818485	112.723797	Jianliyuan	29.848074	112.76888	4.54	Farmlands	6	Direct observation
2006-03-25	J	Sanheyuan	SHY	29.742483	112.59835	SFX	29.576308	112.63071	18.33	Farmlands and woods	U	Direct observation
2006-03-28	R	Sanheyuan	SHY	29.742483	112.59835	Tuanzhou	29.317436	112.8294	49.69	Farmlands	10	Direct observation
2006-04-02	R	Yangbotan	YBT	29.818485	112.723797	Qianzitou	29.838237	112.59488	15.09	Reeds and meadows	5 M	Direct observation
2006-05-09	R	Yangbotan	YBT	29.818485	112.723797	Jinyugou	29.781013	112.59199	15.51	Reeds	20	Direct observation
2006-05-09	R	Yangbotan	YBT	29.818485	112.723797	Qianzitou	29.836889	112.58774	15.83	Woods	5 M	Direct observation
2006-07-03	J	Yangbotan	YBT	29.818485	112.723797	Chenzhou	29.820467	112.77715	10.17	Woods and meadows	15(2 M，10 F，3J)	Direct observation
2006-11-10	R	Yangbotan	YBT	29.818485	112.723797	Xinzhouyuan	29.985467	113.16765	44.98	Farmlands	1 M	Direct observation
2006-12-16	R	Yangbotan	YBT	29.818485	112.723797	Dagonghu	29.86175	112.53758	21.02	Woods	4 M	Direct observation
2007-03-	R	Yangbotan	YBT	29.818485	112.723797	Qianzitou	29.808883	112.59965	14.96	Reeds and	9 M	Direct observation

07										meadows		
2007-03-07	R	Yangbotan	YBT	29.818485	112.723797	Tianxingzhou	29.81825	112.62618	11.74	Meadows and woods	14 M	Direct observation
2007-04-14	R	Sanheyuan	SHY	29.742483	112.59835	Tiaoguan	29.6804	112.6375	7.67	Farmlands	56,36F	Direct observation
2007-06-06	R	Yangbotan	YBT	29.818485	112.723797	Fuxingzhou	29.753377	112.71488	6.88	Reeds	1 M	Direct observation
2007-07-24	J	Sanheyuan	SHY	29.742483	112.59835	Dongsheng Villege	29.714667	112.51112	8.29	Farmlands	2 M	Direct observation
2007-07-24	R	Yangbotan	YBT	29.818485	112.723797	8th Group,Youyi Farm	29.802117	112.81648	6.83	Farmlands	10	Direct observation
2007-07-27	J	Yangbotan	YBT	29.818485	112.723797	Yangzhou	29.793583	112.78577	4.23	Farmlands	1 M	Direct observation
2007-07-27	R	Yangbotan	YBT	29.818485	112.723797	Zhongzhou	29.7661	112.68842	7.59	Woods	4	Direct observation
2007-11-25	J	Sanheyuan	SHY	29.742483	112.59835	NNZ	29.779767	112.48117	11.39	Reeds, woods	9(2 M,4F,3 J)	Direct observation
2008-03-01	R	Sanheyuan	SHY	29.742483	112.59835	Beinianzi	29.7791	112.47455	12.42	Reeds	U	Direct observation
2009-04	J	Zhuzihe	ZZH	29.364575	112.913967	Tuanbei Villege	29.429881	112.81701	11.56	Reeds	10(3 M,7 F)	Direct observation
2009	R	Hongqihu	HQH	29.12078	112.88725	Hanshou	28.89749	112.22272	42.3	Reeds and meadows	U	Hoof prints, fecal pellets
2009-05	J	Sanheyuan	SHY	29.742483	112.59835	SFX	29.554701	112.6288	20.74	Woods，Farmlands	U	Hoof prints, fecal pellets
2009-04	J	Zhuzihe	ZZH	29.364575	112.913967	Tunahuacun	29.3857	112.80441	8.57	Farmlands	4	Hoof prints
2009-01-	R	Sanheyuan	SHY	29.742483	112.59835	Beinianzi	29.777324	112.47301	10.46	Reeds	18	Direct observation

13												
2009-05-09	R	Yangbotan	YBT	29.818485	112.723797	SMNNR	29.779057	112.56089	18.42	Woods，farmlands	1 M	Direct observation
2009-05-30	J	Sanheyuan	SHY	29.742483	112.59835	Tashiyi	29.738979	112.81747	21.88	Farmlands	1 M	Direct observation
2009-06-07	J	Sanheyuan	SHY	29.742483	112.59835	Xinzhoucun	29.985467	113.16765	11.05	Farmlands	1 M	Direct observation
2010-04	J	Zhuzihe	ZZH	29.364575	112.913967	Chaoyanggou Reed Station	29.327892	112.87686	4.91	Reeds	3	Hoof prints
2010-11	J	Zhuzihe	ZZH	29.364575	112.913967	Nanzhou Reed Station	29.311461	112.81183	10.87	Reeds	5	Hoof prints
2010-04	J	Zhuzihe	ZZH	29.364575	112.913967	Dongzhou Reed Station	29.355417	112.88649	2.24	Reeds	20	Hoof prints, hair
2010-07	J	Yangbotan	YBT	29.818485	112.723797	Jiangxinzhou	29.77996	112.48504	15.09	Reeds	28	Hoof prints
2010-11	J	Zhuzihe	ZZH	29.364575	112.913967	Xinsheng Reed Station	29.305133	112.85406	8.19	Reeds	50	Hoof prints, fecal pellets, razing traces
2010-07	J	Yangbotan	YBT	29.818485	112.723797	Hongshan Reed Farm	29.630083	112.91125	33.2	Reeds	1 M	Hoof prints
2010-11	J	Yangbotan	YBT	29.818485	112.723797	Qingshan Reed Station	29.369381	112.94731	2.13	Reeds	1 M	Hoof prints
2010-07	J	Sanheyuan	SHY	29.742483	112.59835	NNZ	29.771737	112.46002	12.19	Reeds	U	Fecal pellets，bedding trace
2010-03	R	Sanheyuan	SHY	29.742483	112.59835	Hongshan Tou	29.644864	112.91602	33.33	Reeds	U	Hoof prints, fecal pellets
2010-06	R	Sanheyuan	SHY	29.742483	112.59835	Xinzhouwaitan	29.765467	112.45182	13.87	Reeds	U	Hoof prints, fecal pellets
2010-02	J	Sanheyuan	SHY	29.742483	112.59835	Yaizhu	29.765436	112.51345	8.03	Farmlands	1	Hoof prints
2010-05	J	Zhuzihe	ZZH	29.364575	112.913967	Zhongzhou	29.766217	112.69359	12.44	Farmlands	1 M	Hoof prints
2010-07	J	Yangbotan	YBT	29.818485	112.723797	Yidui,Huangying	29.844794	112.75314	3.7	Farmlands	U	Hoof prints, fecal pellets

						Farm						
2010-08	J	Yangbotan	YBT	29.818485	112.723797	Qidui, Liugang Farm	29.855064	112.65055	10.47	Farmlands	U	Hoof prints, fecal pellets
2010-08	R	Yangbotan	YBT	29.818485	112.723797	Zhuhu Farm	29.848778	112.64042	11.13	Farmlands	U	Hoof prints, fecal pellets
2010-06	J	Zhuzihe	ZZH	29.364575	112.913967	Tuannan Villege	29.33955	112.82663	8.43	Farmlands	U	Hoof prints ,fecal pellets
2010-07	J	Zhuzihe	ZZH	29.364575	112.913967	Tuanfu Villege	29.339161	112.77465	12.36	Farmlands	U	Hoof prints, fecal pellets
2010-06	J	Zhuzihe	ZZH	29.364575	112.913967	Tunahuacun	29.3857	112.80441	10.72	Farmlands	1	Hoof prints
2010-07	J	Yangbotan	YBT	29.818485	112.723797	Zhongzhou	29.766217	112.69359	7.68	Farmlands	6	Hoof prints, grazing traces
2010-07	R	Yangbotan	YBT	29.818485	112.723797	Huangying Farm	29.844794	112.75314	3.5	Farmlands	9	Hoof prints, fecal pellets
2010-08	J	Sanheyuan	SHY	29.742483	112.59835	SHY	29.761567	112.5659	3.39	Farmlands	6(5 M;1F)	Hoof prints, trampling in rice paddy
2010-07	J	Yangbotan	YBT	29.818485	112.723797	Xiaohe Farm	29.783667	112.60286	14.06	Farmlands	U	Fecal pellets
2010-03	R	Sanheyuan	SHY	29.742483	112.59835	Huagang Villege	29.583442	112.60688	17.39	Farmlands	U	Hoof prints, hair
2010-03	R	Sanheyuan	SHY	29.742483	112.59835	Longxiu Villege	29.569456	112.61548	18.93	Farmlands	U	Hoof prints, hair
2010-03	R	Sanheyuan	SHY	29.742483	112.59835	Qunli Villege	29.53795	112.65794	23.17	Farmlands	U	Hoof prints, fecal pellets
2010-03	R	Sanheyuan	SHY	29.742483	112.59835	Tashiyi	29.740858	112.83848	23.98	Farmlands	U	Hoof prints, fecal pellets
2010-08	J	Yangbotan	YBT	29.818485	112.723797	Jianli	29.855908	112.78432	5.9	Meadows, farmlands	5	Hoof prints, trampling
2010-05	J	Zhuzihe	ZZH	29.364575	112.913967	Zhongzhou Waitan	29.766217	112.69359	12.11	Meadows	2	Hoof prints
2010-03	R	Sanheyuan	SHY	29.742483	112.59835	Jinpeng Villege	29.560017	112.65155	20.69	Woods	U	Hoof prints, fecal pellets
2010-03	R	Sanheyuan	SHY	29.742483	112.59835	Shengfeng Forest Farm	29.574828	112.71297	21.6	Woods	U	Hoof prints, fecal pellets
2010-03	R	Sanheyuan	SHY	29.742483	112.59835	Shishuling	29.55565	112.69063	22.53	Woods	U	Hoof prints, fecal pellets
2010-03	R	Sanheyuan	SHY	29.742483	112.59835	Dongshan Tawn	29.605848	112.77513	23.21	Woods，Farmlands	U	Hoof prints, fecal pellets

2010-03	R	Sanheyuan	SHY	29.742483	112.59835	Honglian Villege	29.563669	112.73437	23.73	Woods，Farmlands	U	Hoof prints, fecal pellets
2010-01-07	R	Yangbotan	YBT	29.818485	112.723797	Heiwawo	29.816162	112.66619	7.96	Reeds and meadows	25	Direct observation
2010-01-23	J	Sanheyuan	SHY	29.742483	112.59835	Beinianzi	29.779878	112.47434	13.82	Reeds	35	Direct observation
2010-01-23	R	Sanheyuan	SHY	29.742483	112.59835	Guafujia	29.777341	112.50981	11.11	Reeds and meadows	19	Direct observation
2010-04-01	J	Zhuzihe	ZZH	29.364575	112.913967	Huanghuajian	29.785564	112.78439	2.03	Reeds	10	Direct observation
2010-04-30	J	Sanheyuan	SHY	29.742483	112.59835	Guafujia	29.771737	112.46002	13.19	Woods	9(4 M，5F)	Direct observation
2010-06-29	J	Yangbotan	YBT	29.818485	112.723797	Yangzhou	29.793583	112.78577	4.89	Farmlands, meadows	1 M	Direct observation
2010-07-27	J	Sanheyuan	SHY	29.742483	112.59835	Sanheyuan Villege	29.738051	112.59199	2.85	Farmlands	54	Direct observation
2010-08-08	R	Yangbotan	YBT	29.818485	112.723797	Jinyugou	29.769189	112.60461	14.51	Woods，farmlands	26(2 M,24F)	Direct observation
2010-10-29	J	Sanheyuan	SHY	29.742483	112.59835	NNZ	29.779063	112.48033	11.22	Reeds and meadows	3F	Direct observation
2010-10-29	R	Sanheyuan	SHY	29.742483	112.59835	Guafujia	29.771455	112.46576	8.93	Reeds and meadows	4M	Direct observation
2011-03	R	Zhuzihe	ZZH	29.364575	112.913967	Bawu Reed Station	29.325806	112.86794	5.27	Reeds	6	Hoof prints
2011-01	J	Zhuczihe	ZZH	29.364575	112.913967	Xinsheng Reed Station	29.305133	112.85406	7.05	Reeds	50	Hoof prints, fecal pellets，trampling
2011-11	J	Yangbotan	YBT	29.818485	112.723797	Hongshan Reed	29.630083	112.91125	23.57	Reeds	2 M	Hoof prints，fecal pellets

						Farm						
2011-03-02	R	Yangbotan	YBT	29.818485	112.723797	Huangying Farm	29.856842	112.67643	8.71	Farmlands	3F	Direct observation
2011-12-29	J	Sanheyuan	SHY	29.742483	112.59835	Guafujia	29.774833	112.52519	7.53	Reeds	8 M	Direct observation
2012	R	Hongqihu	HQH	29.12078	112.88725	ZZH	29.376153	112.93302	20.69	Reeds, swamps	U	Hoof prints, fecal pellets
2012	R	Sanheyuan	SHY	29.742483	112.59835	NNZ	29.780138	112.48363	9.6	Reeds, meadow	U	Hoof prints, fecal pellets
2012	R	Yangbotan	YBT	29.818485	112.723797	Jianli	29.754672	112.71632	16.57	farmlands	U	Hoof prints, fecal pellets
2012	R	Hongqihu	HQH	29.12078	112.88725	Zhongzhou	28.909173	112.1836	24	Woods,farmlands	U	Hoof prints, fecal pellets

Notes:

1 For those data without specific date we only know the year of dispersal occurred.

2 R stands for Routine Survey，which has been routinely carried out in mid of each month；I stands for Instantaneous Survey, a immediately survey carried out when someone reported to the Shishou Milu National Nature reserve about sighting runaway Milu.

3 Distance refers to the linear distance between the start point and destiny of dispersal.

4 M stands for Male; F stands for Female; J stands for Juveniles; U stands for Unknown.
